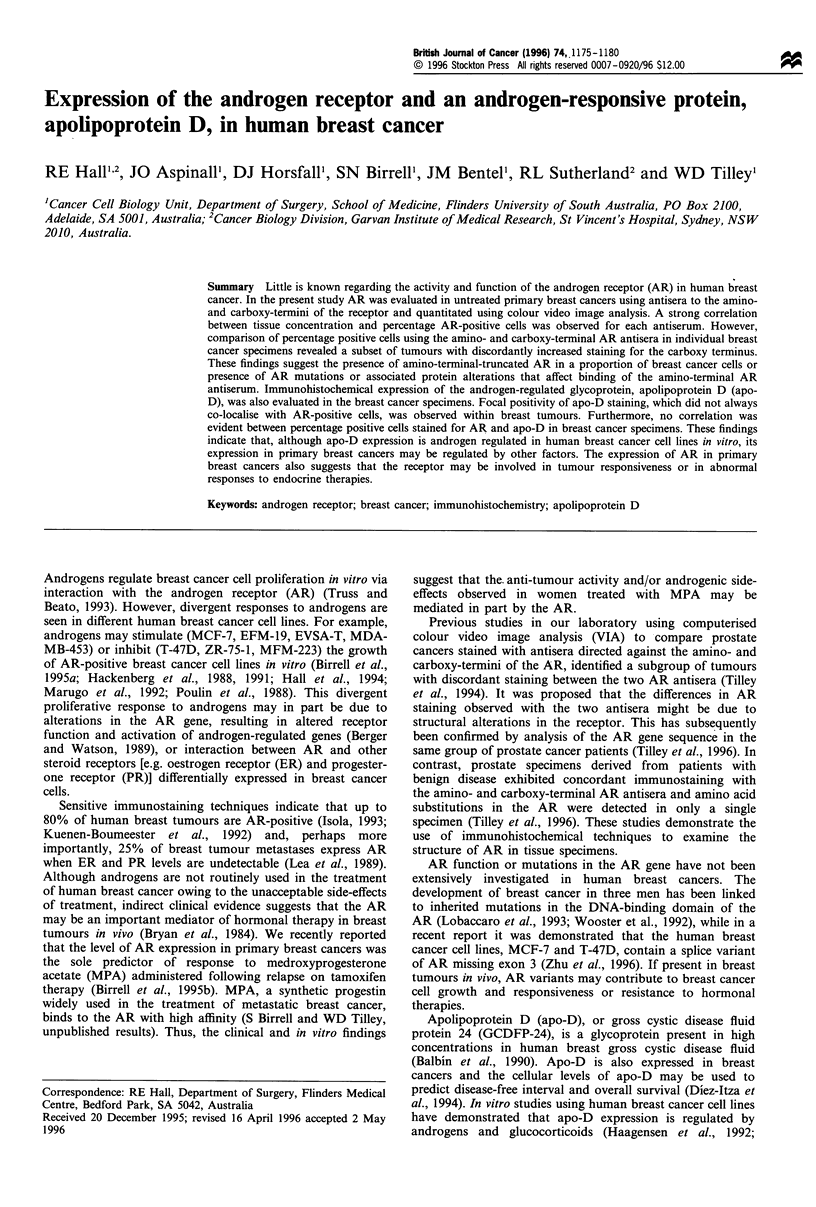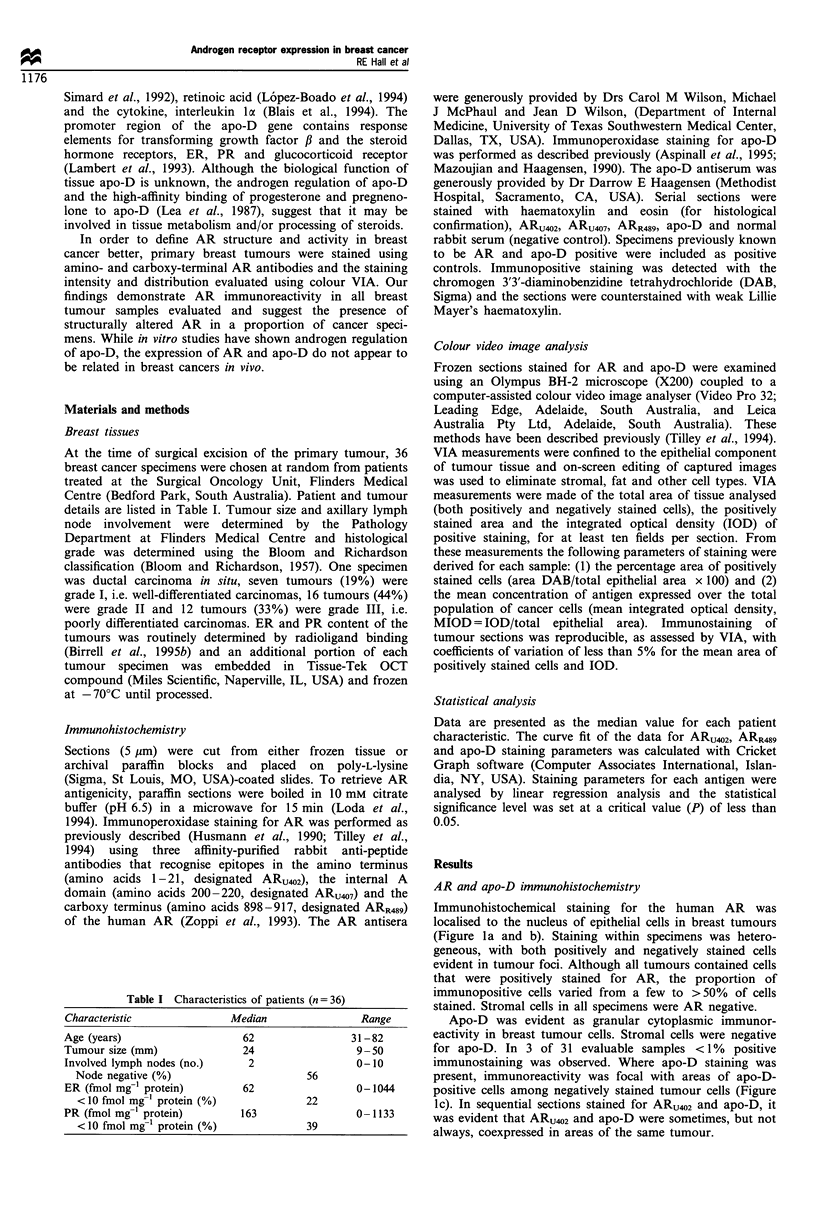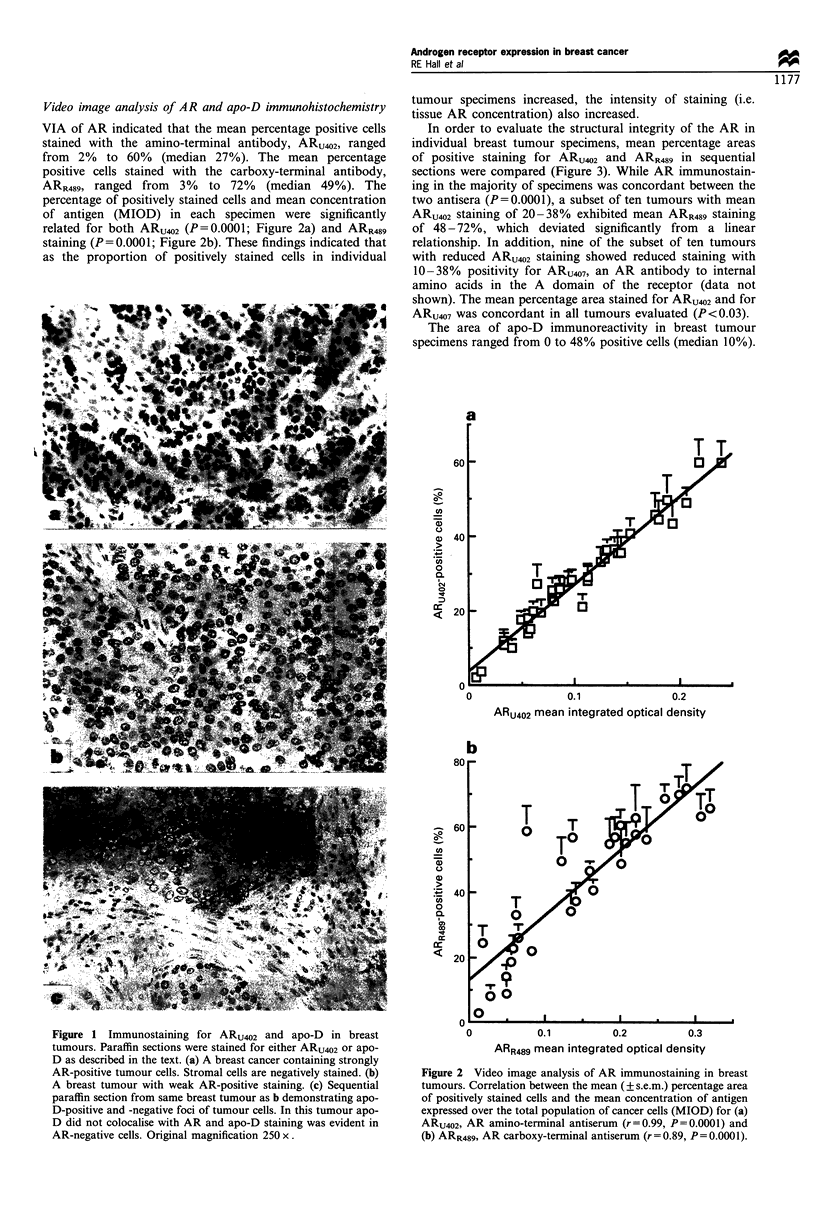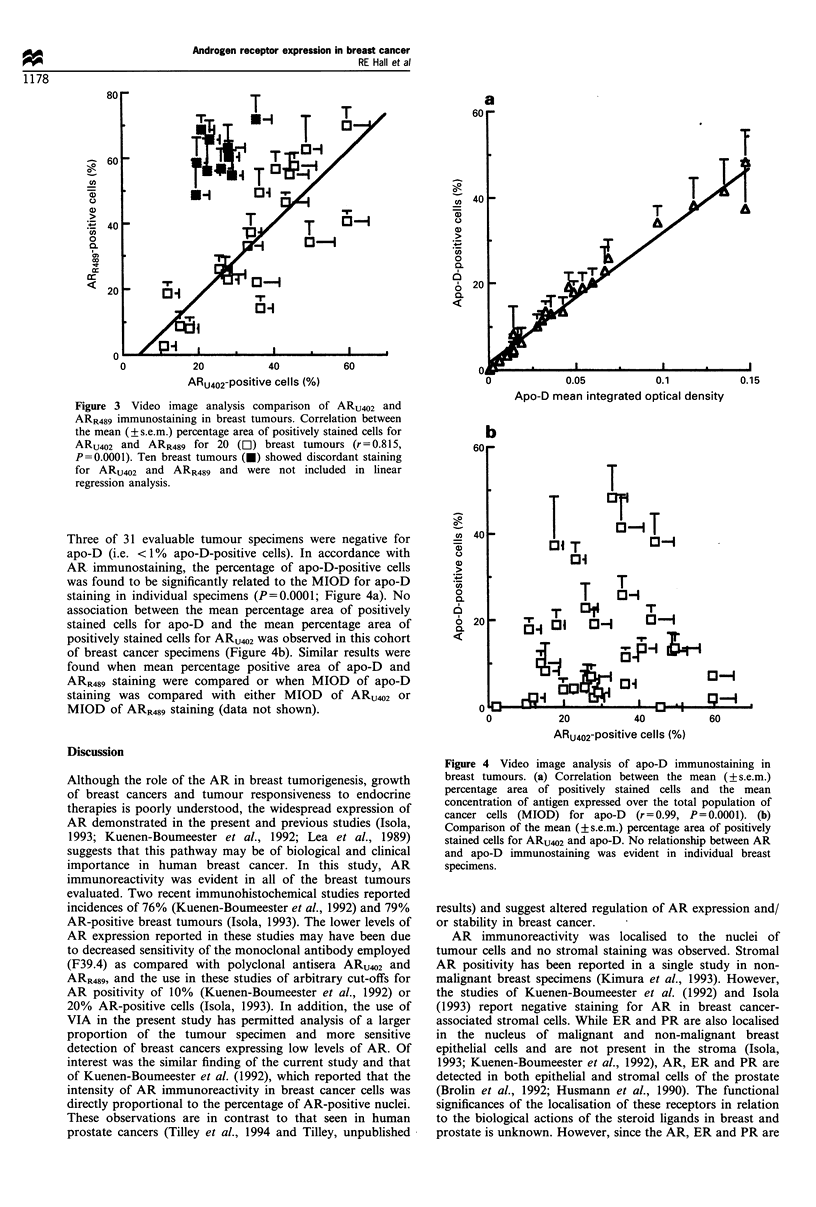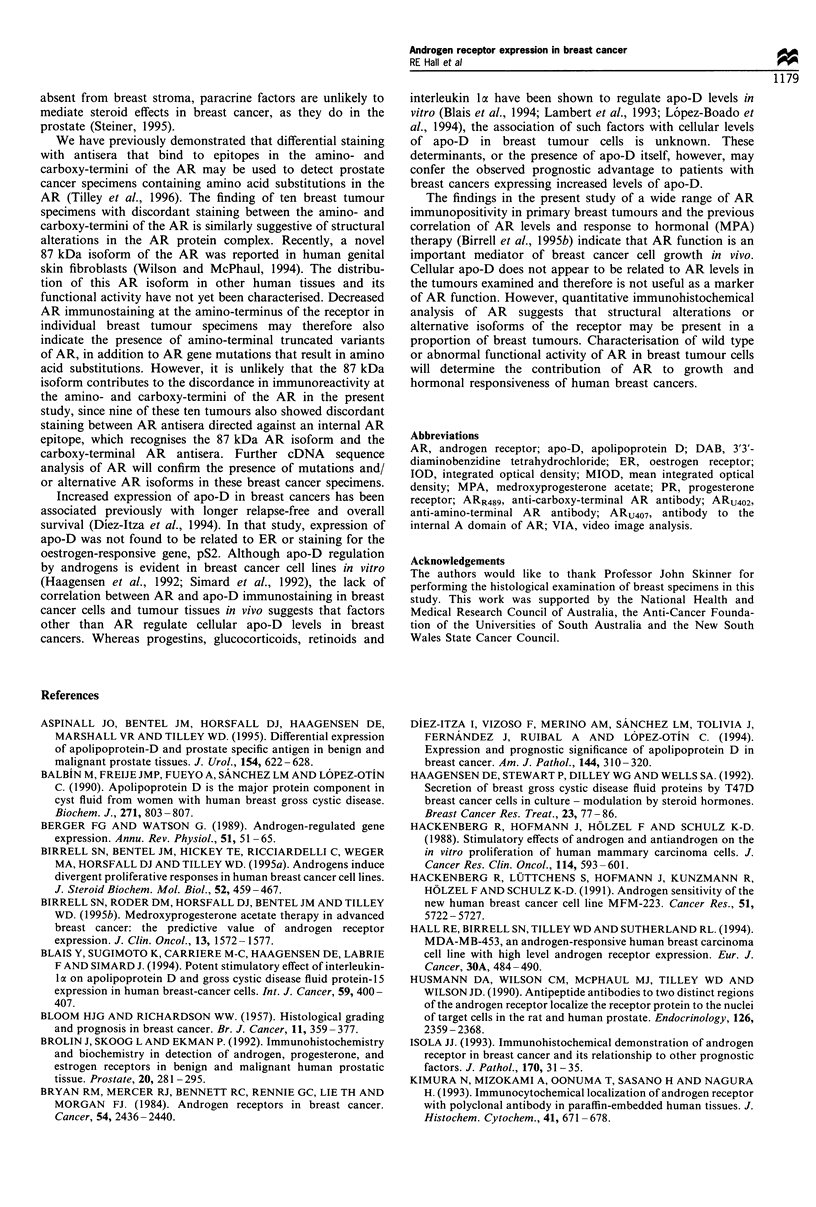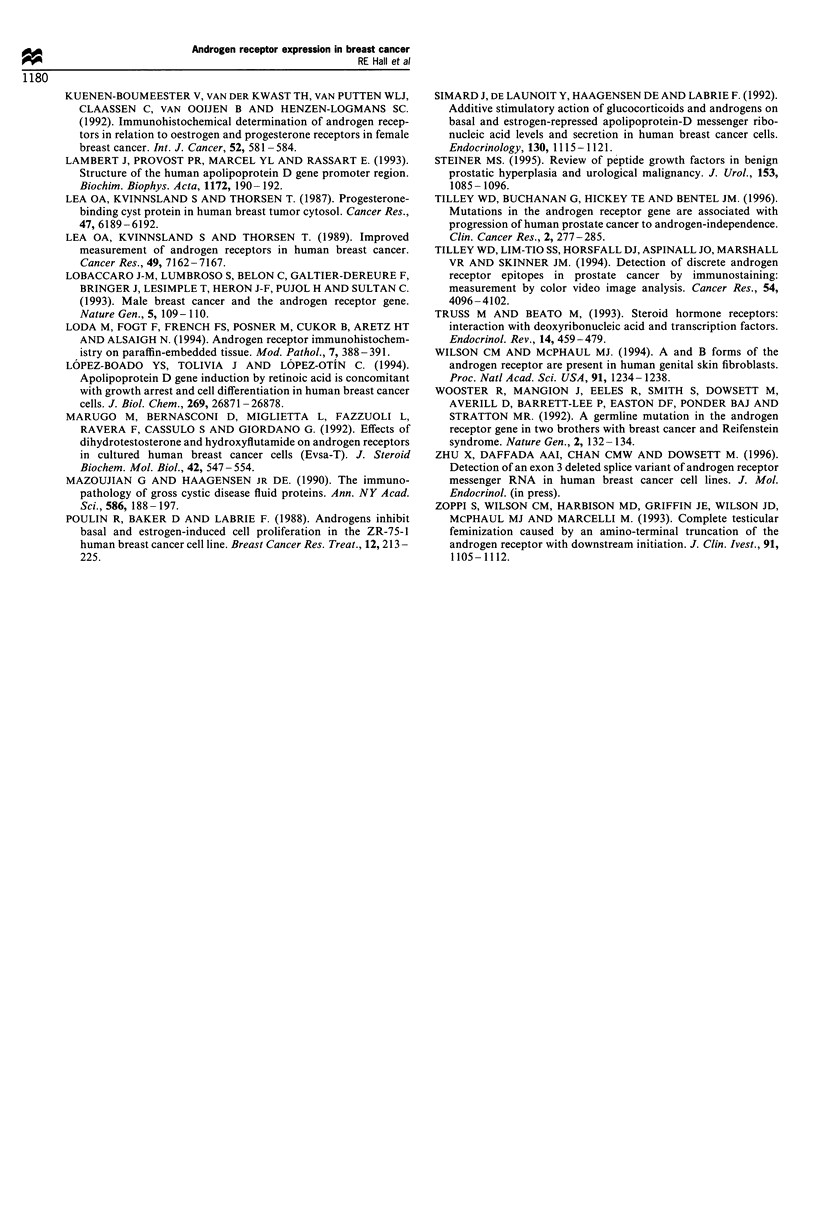# Expression of the androgen receptor and an androgen-responsive protein, apolipoprotein D, in human breast cancer.

**DOI:** 10.1038/bjc.1996.513

**Published:** 1996-10

**Authors:** R. E. Hall, J. O. Aspinall, D. J. Horsfall, S. N. Birrell, J. M. Bentel, R. L. Sutherland, W. D. Tilley

**Affiliations:** Department of Surgery, School of Medicine, Flinders University of South Australia, Adelaide, Australia.

## Abstract

**Images:**


British Journal of Cancer (1996) 74, 1175-1180
© 1996 Stockton Press All rights reserved 0007-0920/96 $12.00

### Expression of the androgen receptor and an androgen-responsive protein, apolipoprotein D, in human breast cancer

RE Hall[1,2], JO Aspinall[1], DJ Horsfall[1], SN Birrell[1], JM Bentel[1], RL Sutherland[2] and WD Tilley[1]

[1]*Cancer Cell Biology Unit, Department of Surgery, School of Medicine, Flinders University of South Australia, PO Box 2100, Adelaide, SA 5001, Australia;* [2]*Cancer Biology Division, Garvan Institute of Medical Research, St Vincent's Hospital, Sydney, NSW 2010, Australia.*

**Summary** Little is known regarding the activity and function of the androgen receptor (AR) in human breast cancer. In the present study AR was evaluated in untreated primary breast cancers using antisera to the amino- and carboxy-termini of the receptor and quantitated using colour video image analysis. A strong correlation between tissue concentration and percentage AR-positive cells was observed for each antiserum. However, comparison of percentage positive cells using the amino- and carboxy-terminal AR antisera in individual breast cancer specimens revealed a subset of tumours with discordantly increased staining for the carboxy terminus. These findings suggest the presence of amino-terminal-truncated AR in a proportion of breast cancer cells or presence of AR mutations or associated protein alterations that affect binding of the amino-terminal AR antiserum. Immunohistochemical expression of the androgen-regulated glycoprotein, apolipoprotein D (apo-D), was also evaluated in the breast cancer specimens. Focal positivity of apo-D staining, which did not always co-localise with AR-positive cells, was observed within breast tumours. Furthermore, no correlation was evident between percentage positive cells stained for AR and apo-D in breast cancer specimens. These findings indicate that, although apo-D expression is androgen regulated in human breast cancer cell lines *in vitro*, its expression in primary breast cancers may be regulated by other factors. The expression of AR in primary breast cancers also suggests that the receptor may be involved in tumour responsiveness or in abnormal responses to endocrine therapies.

**Keywords:** androgen receptor; breast cancer; immunohistochemistry; apolipoprotein D

Androgens regulate breast cancer cell proliferation *in vitro* via interaction with the androgen receptor (AR) (Truss and Beato, 1993). However, divergent responses to androgens are seen in different human breast cancer cell lines. For example, androgens may stimulate (MCF-7, EFM-19, EVSA-T, MDA-MB-453) or inhibit (T-47D, ZR-75-1, MFM-223) the growth of AR-positive breast cancer cell lines *in vitro* (Birrell *et al.*, 1995*a*; Hackenberg *et al.*, 1988, 1991; Hall *et al.*, 1994; Marugo *et al.*, 1992; Poulin *et al.*, 1988). This divergent proliferative response to androgens may in part be due to alterations in the AR gene, resulting in altered receptor function and activation of androgen-regulated genes (Berger and Watson, 1989), or interaction between AR and other steroid receptors [e.g. oestrogen receptor (ER) and progesterone receptor (PR)] differentially expressed in breast cancer cells.

Sensitive immunostaining techniques indicate that up to 80% of human breast tumours are AR-positive (Isola, 1993; Kuenen-Boumeester *et al.*, 1992) and, perhaps more importantly, 25% of breast tumour metastases express AR when ER and PR levels are undetectable (Lea *et al.*, 1989). Although androgens are not routinely used in the treatment of human breast cancer owing to the unacceptable side-effects of treatment, indirect clinical evidence suggests that the AR may be an important mediator of hormonal therapy in breast tumours *in vivo* (Bryan *et al.*, 1984). We recently reported that the level of AR expression in primary breast cancers was the sole predictor of response to medroxyprogesterone acetate (MPA) administered following relapse on tamoxifen therapy (Birrell *et al.*, 1995b). MPA, a synthetic progestin widely used in the treatment of metastatic breast cancer, binds to the AR with high affinity (S Birrell and WD Tilley, unpublished results). Thus, the clinical and *in vitro* findings suggest that the anti-tumour activity and/or androgenic side-effects observed in women treated with MPA may be mediated in part by the AR.

Previous studies in our laboratory using computerised colour video image analysis (VIA) to compare prostate cancers stained with antisera directed against the amino- and carboxy-termini of the AR, identified a subgroup of tumours with discordant staining between the two AR antisera (Tilley *et al.*, 1994). It was proposed that the differences in AR staining observed with the two antisera might be due to structural alterations in the receptor. This has subsequently been confirmed by analysis of the AR gene sequence in the same group of prostate cancer patients (Tilley *et al.*, 1996). In contrast, prostate specimens derived from patients with benign disease exhibited concordant immunostaining with the amino- and carboxy-terminal AR antisera and amino acid substitutions in the AR were detected in only a single specimen (Tilley *et al.*, 1996). These studies demonstrate the use of immunohistochemical techniques to examine the structure of AR in tissue specimens.

AR function or mutations in the AR gene have not been extensively investigated in human breast cancers. The development of breast cancer in three men has been linked to inherited mutations in the DNA-binding domain of the AR (Lobaccaro *et al.*, 1993; Wooster et al., 1992), while in a recent report it was demonstrated that the human breast cancer cell lines, MCF-7 and T-47D, contain a splice variant of AR missing exon 3 (Zhu *et al.*, 1996). If present in breast tumours *in vivo*, AR variants may contribute to breast cancer cell growth and responsiveness or resistance to hormonal therapies.

Apolipoprotein D (apo-D), or gross cystic disease fluid protein 24 (GCDFP-24), is a glycoprotein present in high concentrations in human breast gross cystic disease fluid (Balbín *et al.*, 1990). Apo-D is also expressed in breast cancers and the cellular levels of apo-D may be used to predict disease-free interval and overall survival (Díez-Itza *et al.*, 1994). *In vitro* studies using human breast cancer cell lines have demonstrated that apo-D expression is regulated by androgens and glucocorticoids (Haagensen *et al.*, 1992;

Correspondence: RE Hall, Department of Surgery, Flinders Medical Centre, Bedford Park, SA 5042, Australia
Received 20 December 1995; revised 16 April 1996 accepted 2 May 1996

Simard *et al.*, 1992), retinoic acid (López-Boado *et al.*, 1994) and the cytokine, interleukin 1α (Blais et al., 1994). The promoter region of the apo-D gene contains response elements for transforming growth factor β and the steroid hormone receptors, ER, PR and glucocorticoid receptor (Lambert *et al.*, 1993). Although the biological function of tissue apo-D is unknown, the androgen regulation of apo-D and the high-affinity binding of progesterone and pregnenolone to apo-D (Lea *et al.*, 1987), suggest that it may be involved in tissue metabolism and/or processing of steroids.

In order to define AR structure and activity in breast cancer better, primary breast tumours were stained using amino- and carboxy-terminal AR antibodies and the staining intensity and distribution evaluated using colour VIA. Our findings demonstrate AR immunoreactivity in all breast tumour samples evaluated and suggest the presence of structurally altered AR in a proportion of cancer specimens. While *in vitro* studies have shown androgen regulation of apo-D, the expression of AR and apo-D do not appear to be related in breast cancers *in vivo*.

### Materials and methods

### *Breast tissues*

At the time of surgical excision of the primary tumour, 36 breast cancer specimens were chosen at random from patients treated at the Surgical Oncology Unit, Flinders Medical Centre (Bedford Park, South Australia). Patient and tumour details are listed in Table I. Tumour size and axillary lymph node involvement were determined by the Pathology Department at Flinders Medical Centre and histological grade was determined using the Bloom and Richardson classification (Bloom and Richardson, 1957). One specimen was ductal carcinoma *in situ*, seven tumours (19%) were grade I, i.e. well-differentiated carcinomas, 16 tumours (44%) were grade II and 12 tumours (33%) were grade III, i.e. poorly differentiated carcinomas. ER and PR content of the tumours was routinely determined by radioligand binding (Birrell *et al.*, 1995*b*) and an additional portion of each tumour specimen was embedded in Tissue-Tek OCT compound (Miles Scientific, Naperville, IL, USA) and frozen at −70°C until processed.

### *Immunohistochemistry*

Sections (5 μm) were cut from either frozen tissue or archival paraffin blocks and placed on poly-L-lysine (Sigma, St Louis, MO, USA)-coated slides. To retrieve AR antigenicity, paraffin sections were boiled in 10 mM citrate buffer (pH 6.5) in a microwave for 15 min (Loda *et al.*, 1994). Immunoperoxidase staining for AR was performed as previously described (Husmann *et al.*, 1990; Tilley *et al.*, 1994) using three affinity-purified rabbit anti-peptide antibodies that recognise epitopes in the amino terminus (amino acids 1−21, designated $AR_{U402}$), the internal A domain (amino acids 200−220, designated $AR_{U407}$) and the carboxy terminus (amino acids 898−917, designated $AR_{R489}$) of the human AR (Zoppi *et al.*, 1993). The AR antisera were generously provided by Drs Carol M Wilson, Michael J McPhaul and Jean D Wilson, (Department of Internal Medicine, University of Texas Southwestern Medical Center, Dallas, TX, USA). Immunoperoxidase staining for apo-D was performed as described previously (Aspinall *et al.*, 1995; Mazoujian and Haagensen, 1990). The apo-D antiserum was generously provided by Dr Darrow E Haagensen (Methodist Hospital, Sacramento, CA, USA). Serial sections were stained with haematoxylin and eosin (for histological confirmation), $AR_{U402}$, $AR_{U407}$, $AR_{R489}$, apo-D and normal rabbit serum (negative control). Specimens previously known to be AR and apo-D positive were included as positive controls. Immunopositive staining was detected with the chromogen 3′3′-diaminobenzidine tetrahydrochloride (DAB, Sigma) and the sections were counterstained with weak Lillie Mayer's haematoxylin.

Table I Characteristics of patients ($n = 36$)

| *Characteristic* | *Median* | | *Range* |
|---|---|---|---|
| Age (years) | 62 | | 31−82 |
| Tumour size (mm) | 24 | | 9−50 |
| Involved lymph nodes (no.) | 2 | | 0−10 |
| Node negative (%) | | 56 | |
| ER (fmol $mg^{-1}$ protein) | 62 | | 0−1044 |
| <10 fmol $mg^{-1}$ protein (%) | | 22 | |
| PR (fmol $mg^{-1}$ protein) | 163 | | 0−1133 |
| <10 fmol $mg^{-1}$ protein (%) | | 39 | |

### *Colour video image analysis*

Frozen sections stained for AR and apo-D were examined using an Olympus BH-2 microscope (X200) coupled to a computer-assisted colour video image analyser (Video Pro 32; Leading Edge, Adelaide, South Australia, and Leica Australia Pty Ltd, Adelaide, South Australia). These methods have been described previously (Tilley *et al.*, 1994). VIA measurements were confined to the epithelial component of tumour tissue and on-screen editing of captured images was used to eliminate stromal, fat and other cell types. VIA measurements were made of the total area of tissue analysed (both positively and negatively stained cells), the positively stained area and the integrated optical density (IOD) of positive staining, for at least ten fields per section. From these measurements the following parameters of staining were derived for each sample: (1) the percentage area of positively stained cells (area DAB/total epithelial area ×100) and (2) the mean concentration of antigen expressed over the total population of cancer cells (mean integrated optical density, MIOD = IOD/total epithelial area). Immunostaining of tumour sections was reproducible, as assessed by VIA, with coefficients of variation of less than 5% for the mean area of positively stained cells and IOD.

### *Statistical analysis*

Data are presented as the median value for each patient characteristic. The curve fit of the data for $AR_{U402}$, $AR_{R489}$ and apo-D staining parameters was calculated with Cricket Graph software (Computer Associates International, Islandia, NY, USA). Staining parameters for each antigen were analysed by linear regression analysis and the statistical significance level was set at a critical value ($P$) of less than 0.05.

### Results

### *AR and apo-D immunohistochemistry*

Immunohistochemical staining for the human AR was localised to the nucleus of epithelial cells in breast tumours (Figure 1a and b). Staining within specimens was heterogeneous, with both positively and negatively stained cells evident in tumour foci. Although all tumours contained cells that were positively stained for AR, the proportion of immunopositive cells varied from a few to >50% of cells stained. Stromal cells in all specimens were AR negative.

Apo-D was evident as granular cytoplasmic immunoreactivity in breast tumour cells. Stromal cells were negative for apo-D. In 3 of 31 evaluable samples <1% positive immunostaining was observed. Where apo-D staining was present, immunoreactivity was focal with areas of apo-D-positive cells among negatively stained tumour cells (Figure 1c). In sequential sections stained for $AR_{U402}$ and apo-D, it was evident that $AR_{U402}$ and apo-D were sometimes, but not always, coexpressed in areas of the same tumour.

*Video image analysis of AR and apo-D immunohistochemistry*

VIA of AR indicated that the mean percentage positive cells stained with the amino-terminal antibody, $AR_{U402}$, ranged from 2% to 60% (median 27%). The mean percentage positive cells stained with the carboxy-terminal antibody, $AR_{R489}$, ranged from 3% to 72% (median 49%). The percentage of positively stained cells and mean concentration of antigen (MIOD) in each specimen were significantly related for both $AR_{U402}$ ($P=0.0001$; Figure 2a) and $AR_{R489}$ staining ($P=0.0001$; Figure 2b). These findings indicated that as the proportion of positively stained cells in individual tumour specimens increased, the intensity of staining (i.e. tissue AR concentration) also increased.

In order to evaluate the structural integrity of the AR in individual breast tumour specimens, mean percentage areas of positive staining for $AR_{U402}$ and $AR_{R489}$ in sequential sections were compared (Figure 3). While AR immunostaining in the majority of specimens was concordant between the two antisera ($P=0.0001$), a subset of ten tumours with mean $AR_{U402}$ staining of 20–38% exhibited mean $AR_{R489}$ staining of 48–72%, which deviated significantly from a linear relationship. In addition, nine of the subset of ten tumours with reduced $AR_{U402}$ staining showed reduced staining with 10–38% positivity for $AR_{U407}$, an AR antibody to internal amino acids in the A domain of the receptor (data not shown). The mean percentage area stained for $AR_{U402}$ and for $AR_{U407}$ was concordant in all tumours evaluated ($P<0.03$).

The area of apo-D immunoreactivity in breast tumour specimens ranged from 0 to 48% positive cells (median 10%).

**Figure 1** Immunostaining for $AR_{U402}$ and apo-D in breast tumours. Paraffin sections were stained for either $AR_{U402}$ or apo-D as described in the text. (**a**) A breast cancer containing strongly AR-positive tumour cells. Stromal cells are negatively stained. (**b**) A breast tumour with weak AR-positive staining. (**c**) Sequential paraffin section from same breast tumour as **b** demonstrating apo-D-positive and -negative foci of tumour cells. In this tumour apo-D did not colocalise with AR and apo-D staining was evident in AR-negative cells. Original magnification 250×.

**Figure 2** Video image analysis of AR immunostaining in breast tumours. Correlation between the mean (±s.e.m.) percentage area of positively stained cells and the mean concentration of antigen expressed over the total population of cancer cells (MIOD) for (**a**) $AR_{U402}$, AR amino-terminal antiserum ($r=0.99$, $P=0.0001$) and (**b**) $AR_{R489}$, AR carboxy-terminal antiserum ($r=0.89$, $P=0.0001$).

**Figure 3** Video image analysis comparison of $AR_{U402}$ and $AR_{R489}$ immunostaining in breast tumours. Correlation between the mean (±s.e.m.) percentage area of positively stained cells for $AR_{U402}$ and $AR_{R489}$ for 20 (□) breast tumours ($r=0.815$, $P=0.0001$). Ten breast tumours (■) showed discordant staining for $AR_{U402}$ and $AR_{R489}$ and were not included in linear regression analysis.

Three of 31 evaluable tumour specimens were negative for apo-D (i.e. <1% apo-D-positive cells). In accordance with AR immunostaining, the percentage of apo-D-positive cells was found to be significantly related to the MIOD for apo-D staining in individual specimens ($P=0.0001$; Figure 4a). No association between the mean percentage area of positively stained cells for apo-D and the mean percentage area of positively stained cells for $AR_{U402}$ was observed in this cohort of breast cancer specimens (Figure 4b). Similar results were found when mean percentage positive area of apo-D and $AR_{R489}$ staining were compared or when MIOD of apo-D staining was compared with either MIOD of $AR_{U402}$ or MIOD of $AR_{R489}$ staining (data not shown).

### Discussion

Although the role of the AR in breast tumorigenesis, growth of breast cancers and tumour responsiveness to endocrine therapies is poorly understood, the widespread expression of AR demonstrated in the present and previous studies (Isola, 1993; Kuenen-Boumeester *et al.*, 1992; Lea *et al.*, 1989) suggests that this pathway may be of biological and clinical importance in human breast cancer. In this study, AR immunoreactivity was evident in all of the breast tumours evaluated. Two recent immunohistochemical studies reported incidences of 76% (Kuenen-Boumeester *et al.*, 1992) and 79% AR-positive breast tumours (Isola, 1993). The lower levels of AR expression reported in these studies may have been due to decreased sensitivity of the monoclonal antibody employed (F39.4) as compared with polyclonal antisera $AR_{U402}$ and $AR_{R489}$, and the use in these studies of arbitrary cut-offs for AR positivity of 10% (Kuenen-Boumeester *et al.*, 1992) or 20% AR-positive cells (Isola, 1993). In addition, the use of VIA in the present study has permitted analysis of a larger proportion of the tumour specimen and more sensitive detection of breast cancers expressing low levels of AR. Of interest was the similar finding of the current study and that of Kuenen-Boumeester *et al.* (1992), which reported that the intensity of AR immunoreactivity in breast cancer cells was directly proportional to the percentage of AR-positive nuclei. These observations are in contrast to that seen in human prostate cancers (Tilley *et al.*, 1994 and Tilley, unpublished

**Figure 4** Video image analysis of apo-D immunostaining in breast tumours. (**a**) Correlation between the mean (±s.e.m.) percentage area of positively stained cells and the mean concentration of antigen expressed over the total population of cancer cells (MIOD) for apo-D ($r=0.99$, $P=0.0001$). (**b**) Comparison of the mean (±s.e.m.) percentage area of positively stained cells for $AR_{U402}$ and apo-D. No relationship between AR and apo-D immunostaining was evident in individual breast specimens.

results) and suggest altered regulation of AR expression and/or stability in breast cancer.

AR immunoreactivity was localised to the nuclei of tumour cells and no stromal staining was observed. Stromal AR positivity has been reported in a single study in non-malignant breast specimens (Kimura *et al.*, 1993). However, the studies of Kuenen-Boumeester *et al.* (1992) and Isola (1993) report negative staining for AR in breast cancer-associated stromal cells. While ER and PR are also localised in the nucleus of malignant and non-malignant breast epithelial cells and are not present in the stroma (Isola, 1993; Kuenen-Boumeester *et al.*, 1992), AR, ER and PR are detected in both epithelial and stromal cells of the prostate (Brolin *et al.*, 1992; Husmann *et al.*, 1990). The functional significances of the localisation of these receptors in relation to the biological actions of the steroid ligands in breast and prostate is unknown. However, since the AR, ER and PR are absent from breast stroma, paracrine factors are unlikely to mediate steroid effects in breast cancer, as they do in the prostate (Steiner, 1995).

We have previously demonstrated that differential staining with antisera that bind to epitopes in the amino- and carboxy-termini of the AR may be used to detect prostate cancer specimens containing amino acid substitutions in the AR (Tilley *et al*., 1996). The finding of ten breast tumour specimens with discordant staining between the amino- and carboxy-termini of the AR is similarly suggestive of structural alterations in the AR protein complex. Recently, a novel 87 kDa isoform of the AR was reported in human genital skin fibroblasts (Wilson and McPhaul, 1994). The distribution of this AR isoform in other human tissues and its functional activity have not yet been characterised. Decreased AR immunostaining at the amino-terminus of the receptor in individual breast tumour specimens may therefore also indicate the presence of amino-terminal truncated variants of AR, in addition to AR gene mutations that result in amino acid substitutions. However, it is unlikely that the 87 kDa isoform contributes to the discordance in immunoreactivity at the amino- and carboxy-termini of the AR in the present study, since nine of these ten tumours also showed discordant staining between AR antisera directed against an internal AR epitope, which recognises the 87 kDa AR isoform and the carboxy-terminal AR antisera. Further cDNA sequence analysis of AR will confirm the presence of mutations and/or alternative AR isoforms in these breast cancer specimens.

Increased expression of apo-D in breast cancers has been associated previously with longer relapse-free and overall survival (Díez-Itza *et al*., 1994). In that study, expression of apo-D was not found to be related to ER or staining for the oestrogen-responsive gene, pS2. Although apo-D regulation by androgens is evident in breast cancer cell lines *in vitro* (Haagensen *et al*., 1992; Simard *et al*., 1992), the lack of correlation between AR and apo-D immunostaining in breast cancer cells and tumour tissues *in vivo* suggests that factors other than AR regulate cellular apo-D levels in breast cancers. Whereas progestins, glucocorticoids, retinoids and interleukin 1α have been shown to regulate apo-D levels *in vitro* (Blais *et al*., 1994; Lambert *et al*., 1993; López-Boado *et al*., 1994), the association of such factors with cellular levels of apo-D in breast tumour cells is unknown. These determinants, or the presence of apo-D itself, however, may confer the observed prognostic advantage to patients with breast cancers expressing increased levels of apo-D.

The findings in the present study of a wide range of AR immunopositivity in primary breast tumours and the previous correlation of AR levels and response to hormonal (MPA) therapy (Birrell *et al*., 1995*b*) indicate that AR function is an important mediator of breast cancer cell growth *in vivo*. Cellular apo-D does not appear to be related to AR levels in the tumours examined and therefore is not useful as a marker of AR function. However, quantitative immunohistochemical analysis of AR suggests that structural alterations or alternative isoforms of the receptor may be present in a proportion of breast tumours. Characterisation of wild type or abnormal functional activity of AR in breast tumour cells will determine the contribution of AR to growth and hormonal responsiveness of human breast cancers.

### Abbreviations

AR, androgen receptor; apo-D, apolipoprotein D; DAB, 3′3′-diaminobenzidine tetrahydrochloride; ER, oestrogen receptor; IOD, integrated optical density; MIOD, mean integrated optical density; MPA, medroxyprogesterone acetate; PR, progesterone receptor; $AR_{R489}$, anti-carboxy-terminal AR antibody; $AR_{U402}$, anti-amino-terminal AR antibody; $AR_{U407}$, antibody to the internal A domain of AR; VIA, video image analysis.

### Acknowledgements

The authors would like to thank Professor John Skinner for performing the histological examination of breast specimens in this study. This work was supported by the National Health and Medical Research Council of Australia, the Anti-Cancer Foundation of the Universities of South Australia and the New South Wales State Cancer Council.

### References

ASPINALL JO, BENTEL JM, HORSFALL DJ, HAAGENSEN DE, MARSHALL VR AND TILLEY WD. (1995). Differential expression of apolipoprotein-D and prostate specific antigen in benign and malignant prostate tissues. *J. Urol*., **154,** 622–628.

BALBÍN M, FREIJE JMP, FUEYO A, SÁNCHEZ LM AND LÓPEZ-OTÍN C. (1990). Apolipoprotein D is the major protein component in cyst fluid from women with human breast gross cystic disease. *Biochem. J*., **271,** 803–807.

BERGER FG AND WATSON G. (1989). Androgen-regulated gene expression. *Annu. Rev. Physiol*., **51,** 51–65.

BIRRELL SN, BENTEL JM, HICKEY TE, RICCIARDELLI C, WEGER MA, HORSFALL DJ AND TILLEY WD. (1995*a*). Androgens induce divergent proliferative responses in human breast cancer cell lines. *J. Steroid Biochem. Mol. Biol*., **52,** 459–467.

BIRRELL SN, RODER DM, HORSFALL DJ, BENTEL JM AND TILLEY WD. (1995*b*). Medroxyprogesterone acetate therapy in advanced breast cancer: the predictive value of androgen receptor expression. *J. Clin. Oncol*., **13,** 1572–1577.

BLAIS Y, SUGIMOTO K, CARRIERE M-C, HAAGENSEN DE, LABRIE F AND SIMARD J. (1994). Potent stimulatory effect of interleukin-1α on apolipoprotein D and gross cystic disease fluid protein-15 expression in human breast-cancer cells. *Int. J. Cancer*, **59,** 400–407.

BLOOM HJG AND RICHARDSON WW. (1957). Histological grading and prognosis in breast cancer. *Br. J. Cancer*, **11,** 359–377.

BROLIN J, SKOOG L AND EKMAN P. (1992). Immunohistochemistry and biochemistry in detection of androgen, progesterone, and estrogen receptors in benign and malignant human prostatic tissue. *Prostate*, **20,** 281–295.

BRYAN RM, MERCER RJ, BENNETT RC, RENNIE GC, LIE TH AND MORGAN FJ. (1984). Androgen receptors in breast cancer. *Cancer*, **54,** 2436–2440.

DÍEZ-ITZA I, VIZOSO F, MERINO AM, SÁNCHEZ LM, TOLIVIA J, FERNÁNDEZ J, RUIBAL A AND LÓPEZ-OTÍN C. (1994). Expression and prognostic significance of apolipoprotein D in breast cancer. *Am. J. Pathol*., **144,** 310–320.

HAAGENSEN DE, STEWART P, DILLEY WG AND WELLS SA. (1992). Secretion of breast gross cystic disease fluid proteins by T47D breast cancer cells in culture – modulation by steroid hormones. *Breast Cancer Res. Treat*., **23,** 77–86.

HACKENBERG R, HOFMANN J, HÖLZEL F AND SCHULZ K-D. (1988). Stimulatory effects of androgen and antiandrogen on the *in vitro* proliferation of human mammary carcinoma cells. *J. Cancer Res. Clin. Oncol*., **114,** 593–601.

HACKENBERG R, LÜTTCHENS S, HOFMANN J, KUNZMANN R, HÖLZEL F AND SCHULZ K-D. (1991). Androgen sensitivity of the new human breast cancer cell line MFM-223. *Cancer Res*., **51,** 5722–5727.

HALL RE, BIRRELL SN, TILLEY WD AND SUTHERLAND RL. (1994). MDA-MB-453, an androgen-responsive human breast carcinoma cell line with high level androgen receptor expression. *Eur. J. Cancer*, **30A,** 484–490.

HUSMANN DA, WILSON CM, MCPHAUL MJ, TILLEY WD AND WILSON JD. (1990). Antipeptide antibodies to two distinct regions of the androgen receptor localize the receptor protein to the nuclei of target cells in the rat and human prostate. *Endocrinology*, **126,** 2359–2368.

ISOLA JJ. (1993). Immunohistochemical demonstration of androgen receptor in breast cancer and its relationship to other prognostic factors. *J. Pathol*., **170,** 31–35.

KIMURA N, MIZOKAMI A, OONUMA T, SASANO H AND NAGURA H. (1993). Immunocytochemical localization of androgen receptor with polyclonal antibody in paraffin-embedded human tissues. *J. Histochem. Cytochem*., **41,** 671–678.

KUENEN-BOUMEESTER V, VAN DER KWAST TH, VAN PUTTEN WLJ, CLAASSEN C, VAN OOIJEN B AND HENZEN-LOGMANS SC. (1992). Immunohistochemical determination of androgen receptors in relation to oestrogen and progesterone receptors in female breast cancer. *Int. J. Cancer*, **52**, 581–584.

LAMBERT J, PROVOST PR, MARCEL YL AND RASSART E. (1993). Structure of the human apolipoprotein D gene promoter region. *Biochim. Biophys. Acta*, **1172**, 190–192.

LEA OA, KVINNSLAND S AND THORSEN T. (1987). Progesterone-binding cyst protein in human breast tumor cytosol. *Cancer Res.*, **47**, 6189–6192.

LEA OA, KVINNSLAND S AND THORSEN T. (1989). Improved measurement of androgen receptors in human breast cancer. *Cancer Res.*, **49**, 7162–7167.

LOBACCARO J-M, LUMBROSO S, BELON C, GALTIER-DEREURE F, BRINGER J, LESIMPLE T, HERON J-F, PUJOL H AND SULTAN C. (1993). Male breast cancer and the androgen receptor gene. *Nature Gen.*, **5**, 109–110.

LODA M, FOGT F, FRENCH FS, POSNER M, CUKOR B, ARETZ HT AND ALSAIGH N. (1994). Androgen receptor immunohistochemistry on paraffin-embedded tissue. *Mod. Pathol.*, **7**, 388–391.

LÓPEZ-BOADO YS, TOLIVIA J AND LÓPEZ-OTÍN C. (1994). Apolipoprotein D gene induction by retinoic acid is concomitant with growth arrest and cell differentiation in human breast cancer cells. *J. Biol. Chem.*, **269**, 26871–26878.

MARUGO M, BERNASCONI D, MIGLIETTA L, FAZZUOLI L, RAVERA F, CASSULO S AND GIORDANO G. (1992). Effects of dihydrotestosterone and hydroxyflutamide on androgen receptors in cultured human breast cancer cells (Evsa-T). *J. Steroid Biochem. Mol. Biol.*, **42**, 547–554.

MAZOUJIAN G AND HAAGENSEN JR DE. (1990). The immunopathology of gross cystic disease fluid proteins. *Ann. NY Acad. Sci.*, **586**, 188–197.

POULIN R, BAKER D AND LABRIE F. (1988). Androgens inhibit basal and estrogen-induced cell proliferation in the ZR-75-1 human breast cancer cell line. *Breast Cancer Res. Treat.*, **12**, 213–225.

SIMARD J, DE LAUNOIT Y, HAAGENSEN DE AND LABRIE F. (1992). Additive stimulatory action of glucocorticoids and androgens on basal and estrogen-repressed apolipoprotein-D messenger ribonucleic acid levels and secretion in human breast cancer cells. *Endocrinology*, **130**, 1115–1121.

STEINER MS. (1995). Review of peptide growth factors in benign prostatic hyperplasia and urological malignancy. *J. Urol.*, **153**, 1085–1096.

TILLEY WD, BUCHANAN G, HICKEY TE AND BENTEL JM. (1996). Mutations in the androgen receptor gene are associated with progression of human prostate cancer to androgen-independence. *Clin. Cancer Res.*, **2**, 277–285.

TILLEY WD, LIM-TIO SS, HORSFALL DJ, ASPINALL JO, MARSHALL VR AND SKINNER JM. (1994). Detection of discrete androgen receptor epitopes in prostate cancer by immunostaining: measurement by color video image analysis. *Cancer Res.*, **54**, 4096–4102.

TRUSS M AND BEATO M, (1993). Steroid hormone receptors: interaction with deoxyribonucleic acid and transcription factors. *Endocrinol. Rev.*, **14**, 459–479.

WILSON CM AND MCPHAUL MJ. (1994). A and B forms of the androgen receptor are present in human genital skin fibroblasts. *Proc. Natl Acad. Sci. USA*, **91**, 1234–1238.

WOOSTER R, MANGION J, EELES R, SMITH S, DOWSETT M, AVERILL D, BARRETT-LEE P, EASTON DF, PONDER BAJ AND STRATTON MR. (1992). A germline mutation in the androgen receptor gene in two brothers with breast cancer and Reifenstein syndrome. *Nature Gen.*, **2**, 132–134.

ZHU X, DAFFADA AAI, CHAN CMW AND DOWSETT M. (1996). Detection of an exon 3 deleted splice variant of androgen receptor messenger RNA in human breast cancer cell lines. *J. Mol. Endocrinol.* (in press).

ZOPPI S, WILSON CM, HARBISON MD, GRIFFIN JE, WILSON JD, MCPHAUL MJ AND MARCELLI M. (1993). Complete testicular feminization caused by an amino-terminal truncation of the androgen receptor with downstream initiation. *J. Clin. Ivest.*, **91**, 1105–1112.